# Mitochondrial thiol modification by a targeted electrophile inhibits metabolism in breast adenocarcinoma cells by inhibiting enzyme activity and protein levels

**DOI:** 10.1016/j.redox.2016.01.002

**Published:** 2016-01-08

**Authors:** M. Ryan Smith, Praveen K. Vayalil, Fen Zhou, Gloria A. Benavides, Reena R. Beggs, Hafez Golzarian, Bhavitavya Nijampatnam, Patsy G. Oliver, Robin A.J. Smith, Michael P. Murphy, Sadanandan E. Velu, Aimee Landar

**Affiliations:** a Department of Pathology, University of Alabama at Birmingham, AL, USA; b Center for Free Radical Biology, University of Alabama at Birmingham, AL, USA; c Department of Chemistry, University of Alabama at Birmingham, AL, USA; d Department of Radiation Oncology, University of Alabama at Birmingham, AL, USA; e Department of Chemistry, University of Otago, Dunedin, New Zealand; f MRC Mitochondrial Biology Unit, Cambridge, UK

**Keywords:** ACO, aconitase, BTPP, butyl triphenylphosphonium, DMEM/F12, Dulbecco's modified Eagle's medium/F12, ECAR, extracellular acidification rate, EtOH, ethanol, FBS, fetal bovine serum, FCCP, carbonyl cyanide *p-*trifluoromethoxyphenylhydrazone, GAC, glutaminase C, GAM, glutaminase isoform 3, IBTP, Iodobutyl triphenylphosphonium, KGA, kidney-type glutaminase, MB231, MDA-MB231, MitoE, mitochondria-targeted α-tocopherol, MitoQ, mitochondria-targeted ubiquinol, MRM, multiple reaction monitoring, NEM, N-ethylmaleimide, OCR, oxygen consumption rate, OXPHOS, oxidative phosphorylation, PIC, protease inhibitor cocktail, TBAHS, tetrabutylammonium hydrogen sulfate, TPP, triphenylphosphonium, Bioenergetics, IBTP, Redox signaling, Seahorse extracellular flux analysis, Tricarboxylic acid cycle, Krebs cycle

## Abstract

Many cancer cells follow an aberrant metabolic program to maintain energy for rapid cell proliferation. Metabolic reprogramming often involves the upregulation of glutaminolysis to generate reducing equivalents for the electron transport chain and amino acids for protein synthesis. Critical enzymes involved in metabolism possess a reactive thiolate group, which can be modified by certain oxidants. In the current study, we show that modification of mitochondrial protein thiols by a model compound, iodobutyl triphenylphosphonium (IBTP), decreased mitochondrial metabolism and ATP in MDA-MB 231 (MB231) breast adenocarcinoma cells up to 6 days after an initial 24 h treatment. Mitochondrial thiol modification also depressed oxygen consumption rates (OCR) in a dose-dependent manner to a greater extent than a non-thiol modifying analog, suggesting that thiol reactivity is an important factor in the inhibition of cancer cell metabolism. In non-tumorigenic MCF-10A cells, IBTP also decreased OCR; however the extracellular acidification rate was significantly increased at all but the highest concentration (10 µM) of IBTP indicating that thiol modification can have significantly different effects on bioenergetics in tumorigenic versus non-tumorigenic cells. ATP and other adenonucleotide levels were also decreased by thiol modification up to 6 days post-treatment, indicating a decreased overall energetic state in MB231 cells. Cellular proliferation of MB231 cells was also inhibited up to 6 days post-treatment with little change to cell viability. Targeted metabolomic analyses revealed that thiol modification caused depletion of both Krebs cycle and glutaminolysis intermediates. Further experiments revealed that the activity of the Krebs cycle enzyme, aconitase, was attenuated in response to thiol modification. Additionally, the inhibition of glutaminolysis corresponded to decreased glutaminase C (GAC) protein levels, although other protein levels were unaffected. This study demonstrates for the first time that mitochondrial thiol modification inhibits metabolism via inhibition of both aconitase and GAC in a breast cancer cell model.

## Highlights

• IBTP dependent thiol modification decreases bioenergetics in MB231 and MCF-10A cells.
• IBTP treatment decreases ATP and other adenonucleotides after 1 to 6 days.
• IBTP treatment does not result in overt cellular toxicity.
• IBTP treatment decreases levels of bioenergetically-linked metabolites.
• IBTP treatment decreases aconitase activity and glutaminase protein levels.

## Graphical abstract

**Figure:**
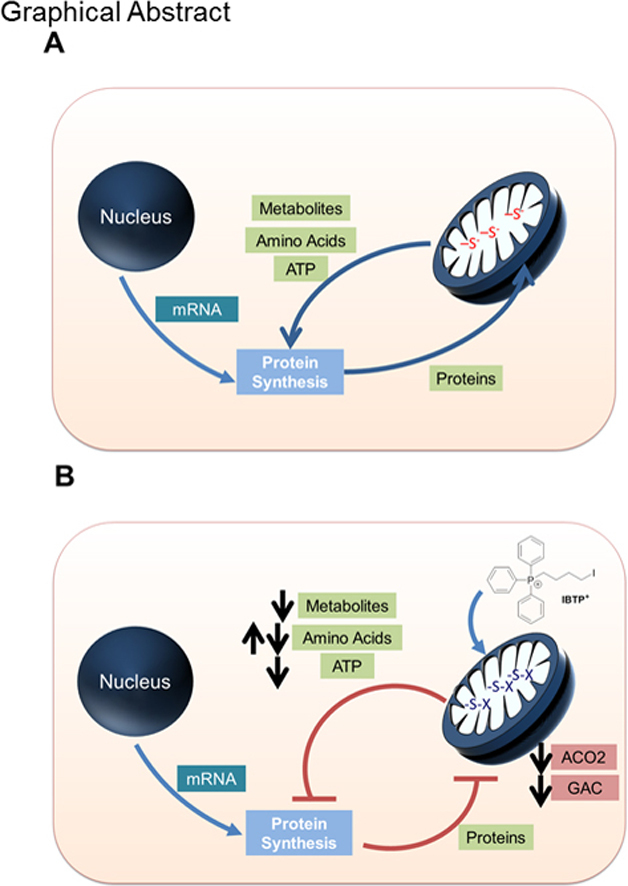

## 1 Introduction

One of the hallmarks of cancer cell transformation is the dysregulation of energetic pathways [1]. This process, known as “metabolic reprogramming,” involves an increased reliance on glycolysis independent of oxygen levels to not only provide ATP, but also to provide intermediates for new lipids, amino and nucleic acids needed for rapid cell proliferation. In addition, upregulation of glutaminolysis is observed in many diverse cancer types including breast cancer, glioblastoma multiforme, and pancreatic cancer [2], [3], [4], [5], [6], [7]. Glutaminolysis is an anaplerotic pathway that replenishes the Krebs cycle by the conversion of glutamine to α-ketoglutarate, thereby providing reducing equivalents to drive electron transport. Glutaminolysis is also important in providing intermediates necessary for the rapid synthesis of amino acids and glutathione [8], [9]. In cancer cells, upregulation of glutaminolysis within the mitochondrion allows for generation of ATP with high efficiency, while also generating substrates required in protein synthesis serving as a complement to glycolysis for biomass synthesis and energy production [7].

Cells that are dependent on glutamine for proliferation are termed “glutamine addicted,” and this feature presents an attractive therapeutic target, due to the fact that most normal non-tumorigenic cells are not glutamine addicted [10]. A key mediator of glutamine-addiction, the enzyme glutaminase C (GAC), is upregulated as a result of oncogenic gene transcription, and has recently become a widely-recognized target for the development of novel therapeutics [11], [12], [13]. However, current GAC inhibitors suffer from relatively low solubility, low-affinity and high toxicity [14], [15], [16], making novel inhibitors of glutaminolysis attractive for development. Of note, recent studies have also indicated that GAC possesses a reactive cysteine thiolate, which have been characterized as redox switches for other proteins involved in pathways such as metabolism and cellular proliferation [17], [18].

In order to inhibit metabolism within the mitochondrion, it is necessary to design agents which not only enter the organelle, but an also inhibit one or more enzymes within the pathway. An effective method to target compounds to the mitochondrion is by coupling to a lipophilic cationic moiety, such as triphenylphosphonium (TPP), which allows the conjugated product to accumulate within the mitochondrion 100–500 fold based on mitochondrial membrane potential [19], [20]. TPP has been used to target compounds such as doxorubicin, coenzyme Q, and electrophiles to the mitochondria to enhance cell death, protect against oxidative stress, or modulate antioxidant responses, respectively [21], [22], [23]. Iodobutyl triphenylphosphonium (IBTP) is a mitochondria-targeted electrophile which was originally developed as a research tool to probe the status of reduced thiols in isolated mitochondria, cell culture models, and isolated tissues from animal models of oxidative stress [24], [25]. In a previous study, we reported that IBTP affected cellular function by inhibiting Nrf2-dependent antioxidant responses in endothelial cells [26]. More recently, we showed that IBTP inhibited overall metabolism in breast cancer cells after a short (4h) exposure, and also prevented cell migration and adhesion [27]. Due to the soft electrophilic nature of IBTP, this compound forms a covalent adduct with specific cysteinyl thiol groups of proteins, many of which play a central role in cell metabolism [24], [25], [28], [29].

In this study, we sought to determine the mechanisms by which mitochondrial thiol modification inhibits metabolism in MDA-MB-231 (MB231) cells. MB231 cells represent a prototype of metabolically reprogrammed glutamine-dependent cancer cells. These “triple-negative” cells are characterized by lacking estrogen, progesterone, and Her2/neu receptors. In addition, these cells are rapidly proliferating, tumorigenic and metastatic, making them an ideal model for aggressive cancer cell types. Herein, we show that mitochondrial thiol modification blocks energy production by decreasing protein levels and activity, depleting Krebs cycle intermediates and inhibiting oxidative phosphorylation, ultimately decreasing ATP levels. Furthermore, our data suggest that likely mechanisms of IBTP inhibition of metabolism include decreasing enzyme activity and/or decreasing protein levels.

## 2 Experimental

### 2.1 Materials

All chemicals were of analytical grade and purchased from Sigma-Aldrich (St. Louis, MO) unless otherwise noted. IBTP and BTPP were prepared as previously described [24]. Anti-TPP antiserum was prepared as previously described [24]. Monoclonal [Abcam (Cambridge, MA), ab156876] or polyclonal [Proteintech (Chicago, IL), 23549-1-AP] anti-glutaminase antibodies were used as indicated in the. Other antibodies used were anti-β-actin [Cell Signaling (Beverly, MA], polyclonal anti-citrate synthetase [Abcam, ab96600], anti-complex IV subunit 2 [Life Technologies (Grand Island, NY); 20E8C12], affinity purified anti-aconitase (a generous gift from Dr. Scott Ballinger at UAB, prepared as described in [30]). The anti-aconitase antibody recognizes both mitochondrial and cytosolic aconitase (ACO2 and ACO1, respectively) isoforms in human breast cancer cells at distinguishable molecular weights (unpublished observations Smith, Zhou, Landar).

### 2.2 Cell culture and treatments

MDA-MB-231 (MB231) human breast adenocarcinoma cells were a generous gift from Dr. Danny Welch, and were originally obtained from ATCC (Manassas, VA). MB231 were cultured in Dulbecco's modified Eagle's medium/F12 (DMEM/F12) (Mediatech, Manassas, VA) supplemented with 10% fetal bovine serum (FBS; Atlanta Biologicals, Atlanta, GA). Cultures were maintained in 5% CO2 and humidified in a 37 °C incubator. Cells were plated at a density of 2**×**10^5^ cells/well in 6-well plates and cultured for 48 h, unless otherwise indicated. The medium was changed to low FBS medium (0.5%) for 16 h prior to treatments. Cells were treated with EtOH vehicle, IBTP or BTPP in 2 mL of fresh medium at concentrations and times indicated in each experiment. MCF-10A human breast epithelial cells were a generous gift from Dr. Danny Welch, and were originally obtained from ATCC (Manassas, VA). MCF10A were cultured in Dulbecco's modified Eagle's medium/F12 (DMEM/F12) (Mediatech, Manassas, VA) supplemented with BulletKit™ (Lonza; Basel Switzerland). Cultures were maintained in 5% CO2 and humidified in a 37 °C incubator. Cells were plated at a density of 2**×**10^5^ cells/well in 6-well plates and cultured for 48h, unless otherwise indicated. The medium was changed to low supplement medium (0.5%) for 16h prior to treatments. Cells were treated with EtOH vehicle, IBTP or BTPP in 2 mL of fresh medium at concentrations and times indicated in each experiment.

### 2.3 Immunoblot analysis

Cells were washed with PBS and lysed in Lysis Buffer [10 mM Tris–HCl, pH 7.4, 1% Triton X-100 containing protease inhibitor cocktail (PIC; Roche)]. Soluble proteins were resolved using SDS-PAGE and transferred to nitrocellulose membranes (Bio-Rad). Protein levels were quantified using the method of DC-Lowry (Bio-Rad), and equivalent amounts of protein were loaded. Uniform protein loading was confirmed using Ponceau S staining of membranes and showed no significant differences in protein levels among samples. Membranes were blocked in 5% milk (*w*/*v*) in Tris Buffered Saline (pH 7.4) containing 0.05% Tween 20 (TBS-T), and incubated with antibodies [polyclonal anti-glutaminase (1:10,000), monoclonal anti-GAC/KGA/GAM (1:3000), anti-citrate synthase (1:1000), anti-complex IV subunit 2 (1:1000), anti-β-actin (1:1000), anti-aconitase 1 (1:5000), and anti-TPP (1:10,000) primary antisera]. All antibodies were incubated overnight at 4 °C, except anti-TPP which was incubated for 3h at ambient temperature. Membranes were washed, incubated with HRP-conjugated anti-rabbit or anti-mouse IgG secondary antibody, and developed using SuperSignal West Dura chemiluminescence substrate (Pierce). Images were obtained using an Amersham™ Imager 600 (GE Healthcare Biosciences; Pittsburg PA).

### 2.4 Metabolic rate assessment

To measure mitochondrial metabolism in intact MB231 cells, an XF96 Extracellular Flux Analyzer was used with a mitochondrial “stress test” [32]. The optimal seeding density of MB231 was determined to be 15,000 cells per well and MCF10A were determined to be 40,000 cells per well. The mitochondrial stress test was performed as described previously [33], [34], [35]. Concentrations of oligomycin, carbonyl cyanide *p-*trifluoromethoxyphenylhydrazone (FCCP), and antimycin A were optimized prior to the assay for each cell line. In our calculations, the oligomycin-insensitive oxygen consumption rate (OCR) was attributed to proton leak. To determine the effects of IBTP at a later time point on mitochondrial metabolism, cells were cultured as previously described. The cells were treated with the indicated concentrations of IBTP or BTPP for 24 h in 0.5% FBS-containing or 0.5% nutrient-containing medium. After the incubation, the cells were harvested immediately by trypsinization and replated at a density of 15,000 or 40,000 cells per well into XF96 plates for an additional 24 h in complete medium containing 10% FBS or 10% nutrients (total 48 h). After incubation, the medium was removed and replaced with XF assay medium (DMEM, containing 15 mM glucose, 2.5 mM glutamine, 0.5 mM pyruvate without bicarbonate) and equilibrated 1h before OCR measurement. Both OCR and extracellular acidification rate (ECAR) measurements were normalized to number of cells plated per well (15,000 or 40,000 cells respectively) because total protein per well was below limit of detection for the MB231 cells.

### 2.5 Targeted metabolomic analysis

Cells were cultured as described above and treated with vehicle, IBTP (10 µM) or BTPP (10 µM), for 24 h. Cells were then washed with PBS and lysed with 500 µL of filtered HPLC grade methanol. The wells were scraped and 2 wells pooled and collected for sample analysis in a glass tube. The methanol extracts were brought to the Targeted Metabolomics and Proteomics Laboratory where they were stored at −80° until analyzed. The samples were taken to dryness under a stream of nitrogen gas. Samples were reconstituted by the addition of 50 µL of 5% acetic acid followed by vortex mixing. 50 µL of Amplifex Keto Reagent Kit (Applied Biosystems part number 4465962) was then added to each sample with vortex mixing. Tubes were capped and placed on a rotary shaker. After 90 min, samples were removed, vortexed a final time, and placed under a stream of nitrogen gas until dry. Final reconstitution was in 500 µL Milli-Q water. Separation of the Krebs Cycle metabolites was performed on a Synergi 4µ Hydro-RP 80A column (Phenomenex part number 00G-4375-B0) with dimensions of 2.0**×**250 mm. Mobile phase A was water+0.1% formic acid, mobile phase B was methanol+0.1% formic acid. Flow rate was 0.25 mL/min with the following gradient: 0 min=5% B, 1 min=5% B, 7 min=40% B, 10 min=100% B, 10.5 min=5% B, 15 min=stop. Injection volume was 10 µL.

### 2.6 Nucleotide extraction

All nucleotide samples were prepared by modifying a method previously described [31]. Briefly, an aliquot of protein-precipitated lysate was obtained from MB231 cells after the indicated treatments by scraping the cells after addition of 5% perchloric acid. The lysate was centrifuged (20,800*g* for 5 min at 4 °C) and the supernatant was transferred into an Eppendorf tube and neutralized by precipitating ClO_4_^−^ with K_2_HPO_4_. The suspension was vortexed, kept on ice for 10 min and then centrifuged (as above) to remove salt. The supernatant was used immediately or stored at −80 °C until analysis. The precipitated protein pellet was stored and later resuspended in 300 µL of 1N NaOH and protein concentration was determined by DC-Lowry with BSA as a standard.

### 2.7 HPLC separation and measurement of adenine nucleotides

Nucleotide analysis was performed as previously described [31]. The HPLC consisted of a Gold HPLC model equipped with System Gold 168 Detector and System Gold Autosampler 507 from Beckman Coulter. The analytical column was a Supelcosil LC-18-T, (150 mm×4.6 mm internal diameter, particle size 3 µm) from Sigma-Aldrich. Analytical runs were processed by 32 Karat Software (version 8.0) also from Beckman Coulter. The chromatographic separation was performed at ambient temperature with gradient elution. The mobile-phase flow rate was set at 1 ml/min and consisted of 65 mM potassium phosphate buffer and 4 mM tetrabutylammonium hydrogen sulfate (TBAHS) adjusted to pH 6.0 with orthophosphoric acid (Buffer A) and 30% MeOH in 65 mM potassium phosphate buffer with 4 mM TBAHS adjusted to pH 6.0 with orthophosphoric acid (Buffer B). The buffers were delivered in a gradient as follows: 0–2 min, 30% Buffer B, 2–16 min to 90% Buffer B; 16–20 min to 90% Buffer B; 20–21 min returned to 30% Buffer B; and 21–24 min 30% Buffer B using an 2 min equilibration between injections. The injection volume was 10 µL. Nucleotides were monitored at 254 and 262 nm. Standard AMP, ADP, ATP and NAD^+^ were dissolved in Buffer A at concentrations from 2 to 100 µM to create a standard curve. Standards were not filtered prior to injection. Experimental samples were prepared as follows: a volume of 150 µL of nucleotide extract suspension was mixed 1:1 with 150 µL of Buffer A and filtered prior to injection in HPLC.

### 2.8 ATP luminescence assay

Relative ATP levels were determined using the luminescence based ATPLite™ assay (Perkin-Elmer, Waltham, MA) and measured on a TopCount NXT microplate scintillation and luminescence counter (Packard, Meriden, CT). In brief, cells were cultured as described above. The cells were then treated with IBTP (0.01–10 µM), BTPP (0.01–10 µM) or vehicle (EtOH) for 24 h, and media replaced with 10% FBS DMEM/F12 for an additional 24 h, to allow cells to recover. For experiments exceeding 24 h, cells were treated as described above, and media was replaced every 48 h with 10% FBS DMEM/F12 until day 6. To measure ATP, 1.5 mL media was decanted and 250 µL of the ATPLite™ Lysis Buffer was added to the remaining 0.5 mL media in each well. Samples were mixed on an orbital shaker for 5min, samples were dark-adapted, and luminescence was measured. In order to determine protein concentration, a parallel well was lysed in 80 µL of lysis buffer as described above, and protein measured by DC Lowry.

### 2.9 Aconitase activity assay

The method was performed as previously described [32], [33]. In brief, cells were cultured as described above. The cells were then treated with IBTP (5–10 µM), BTPP (5–10 µM), or vehicle (EtOH) for 24 h. The cells were then washed with PBS and lysed with Lysis buffer [50 mM Tris, pH 7.4, 0.6 mM MnCl_2_ 2 mM sodium citrate, and 0.5% Triton X100]. 50 µg of protein was loaded onto a native gel [8% acrylamide, 132 mM Tris, 132 mM borate, 3.5 mM citrate] and a stacker [4% acrylamide, 67 mM Tris base, 67 mM borate, 3.6 mM citrate]. The running buffer contains 25 mM Tris, pH 8, 192 mM glycine, 3.6 mM citrate. Samples contain cell lysates, 25 mM Tris-Cl, pH 8, 0.025% sodium bromophenol blue, 10% glycerol. Electrophoresis was carried out at 180 V at 4 °C for 3 h. The gels were then incubated with staining solution [100 mM Tris, pH 8, 1 mM NADP^+^, 2.5 mM cis-aconitic acid, 5 mM MgCl_2_, 1.2 mM MTT, 0.3 mM phenazine methosulfate, and 5 U/mL isocitrate dehydrogenase (Sigma #i2002) for 30 min in the dark at 37 °C. The reaction was then stopped by 2 washes of 100 mM Tris, pH 8. Images were then taken using an Amersham™ Imager 600 (GE Healthcare Biosciences; Pittsburg PA)

### 2.10 Cell survival assessment

Cell survival was determined by trypan blue exclusion. Briefly, cells were seeded at 200,000 cells/well in a 6 well plate and incubated at 37° for 48 h in DMEM/F12 10% FBS. The cells were then transferred to 2 mL 0.5% FBS DMEM/F12 media overnight. The cells were then treated 10 µM IBTP, 10 µM BTPP or Vehicle (EtOH) for 24 h. The media was then replaced with 10% FBS DMEM/F12 and was replaced every 48 h until day 6. At each of the indicated time points cells were trypsinized using 0.5 mL of 0.05% Trypsin EDTA, and neutralized via 0.5 mL of 10% FBS DMEM/F12 media. Cells were then counted via a Biorad TC20™ automated cell counter and cell survival was determined using Trypan Blue Due 0.4% (Bio-rad, Hercules, CA).

### 2.11 Cell Proliferation Assessment

Cell proliferation was determined by counting the cells using a hemocytometer. Briefly, cells were seeded at 200,000 cells/well in a 6 well plate and incubated at 37° for 48 h in DMEM/F12 10% FBS. The cells were then transferred to 2 mL 0.5% FBS DMEM/F12 media overnight. The cells were then treated 10 µM IBTP, 10 µM BTPP or Vehicle (EtOH) for 24 h. The media was then replaced with 10% FBS DMEM/F12 and was replaced every 48 h until day 6. At each of the indicated time points cells were trypsinized using 0.5 mL of 0.05% Trypsin EDTA, and neutralized via 0.5 mL of 10% FBS DMEM/F12 media. Cells were then counted via a Biorad TC20™ automated cell counter (Bio-rad, Hercules, CA)

### 2.12 Statistical analysis

Results are reported as mean±SEM, and *n*=3 or more determinations, or otherwise as indicated in the. Data were analyzed by unpaired *t*-tests or one-way analysis of variance (ANOVA) followed by Tukey post-hoc analysis using GraphPad Software. The minimum level of significance was set at *p*<0.05.

## 3 Results

### 3.1 Mitochondrial thiol modification affects bioenergetics

**Fig. 1:**
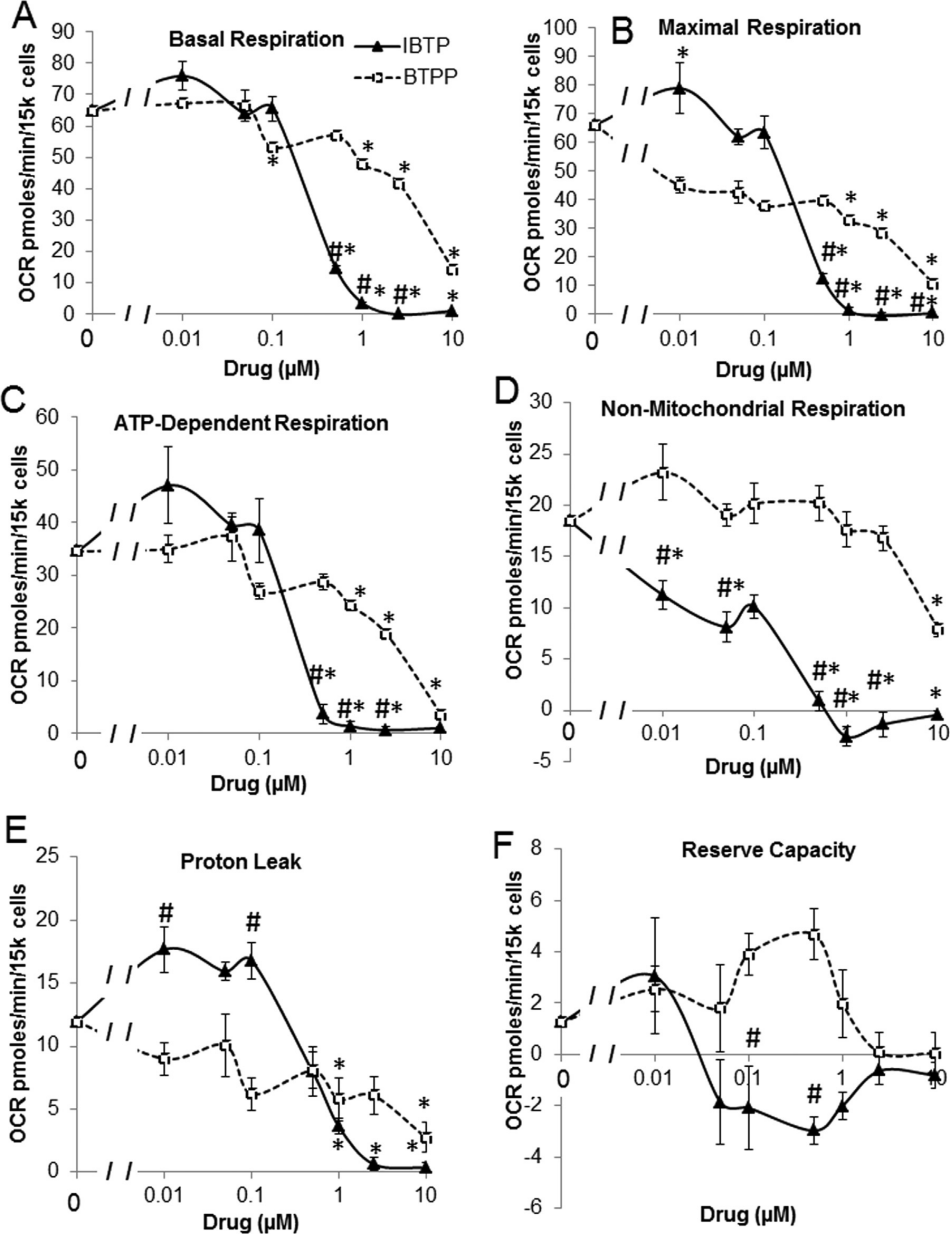
Mitochondrial stress test dose response curves. MB231 cells were treated with vehicle (EtOH), IBTP (0.01–10 µM), or BTPP (0.01–10 µM) for 24 h and a mitochondrial stress test was performed as described in the Methods. Panel A: Dose response of IBTP or BTPP on basal respiration. Panel B: Dose response of IBTP or BTPP on maximal respiration. C: Dose response of IBTP or BTPP on ADP-dependent respiration D: Dose response of IBTP or BTPP on non-mitochondrial respiration. E: Dose Response of IBTP or BTPP on proton leak F: Dose response of IBTP or BTPP on reserve capacity. Values are mean±SEM obtained from 11 to 24 wells in three separate experiments; *P<0.05 compared to vehicle; # *P*<0.05 compared to BTPP.

IBTP forms covalent mitochondrial protein adducts, and at concentrations of 5 or 10 µM for 4 h, inhibits bioenergetics in MB231 cells [27]. However, the effects of lower doses of IBTP for longer treatment times on bioenergetics have not been fully explored in these cells. Therefore, extracellular flux analysis was used to measure glycolytic and mitochondrial bioenergetic parameters. MB231 cells were treated for 24 h with a dose range (0.01–10 µM) of IBTP, a non-electrophilic compound BTPP, or vehicle (EtOH). Bioenergetic parameters were measured using a mitochondrial stress test, and changes in the oxygen consumption rates are shown in Fig. 1. Mitochondrial thiol modification by IBTP was found to decrease basal, ATP-dependent respiration, maximal, and non-mitochondrial respiration bioenergetic parameters, excluding reserve capacity and proton leak, from 0.5 µM to 10 µM compared to vehicle (indicated as 0 µM) and also exhibited differential effects from BTPP (Fig. 1A–D). BTPP also decreased basal, non-mitochondrial respiration, ATP-dependent respiration, maximal respiration, and proton leak excluding reserve capacity, but at higher concentrations than those seen with IBTP, (1–10 µM); however BTPP also exhibited unique changes proton leak when compared to IBTP (0.1 µM).

**Fig. 2:**
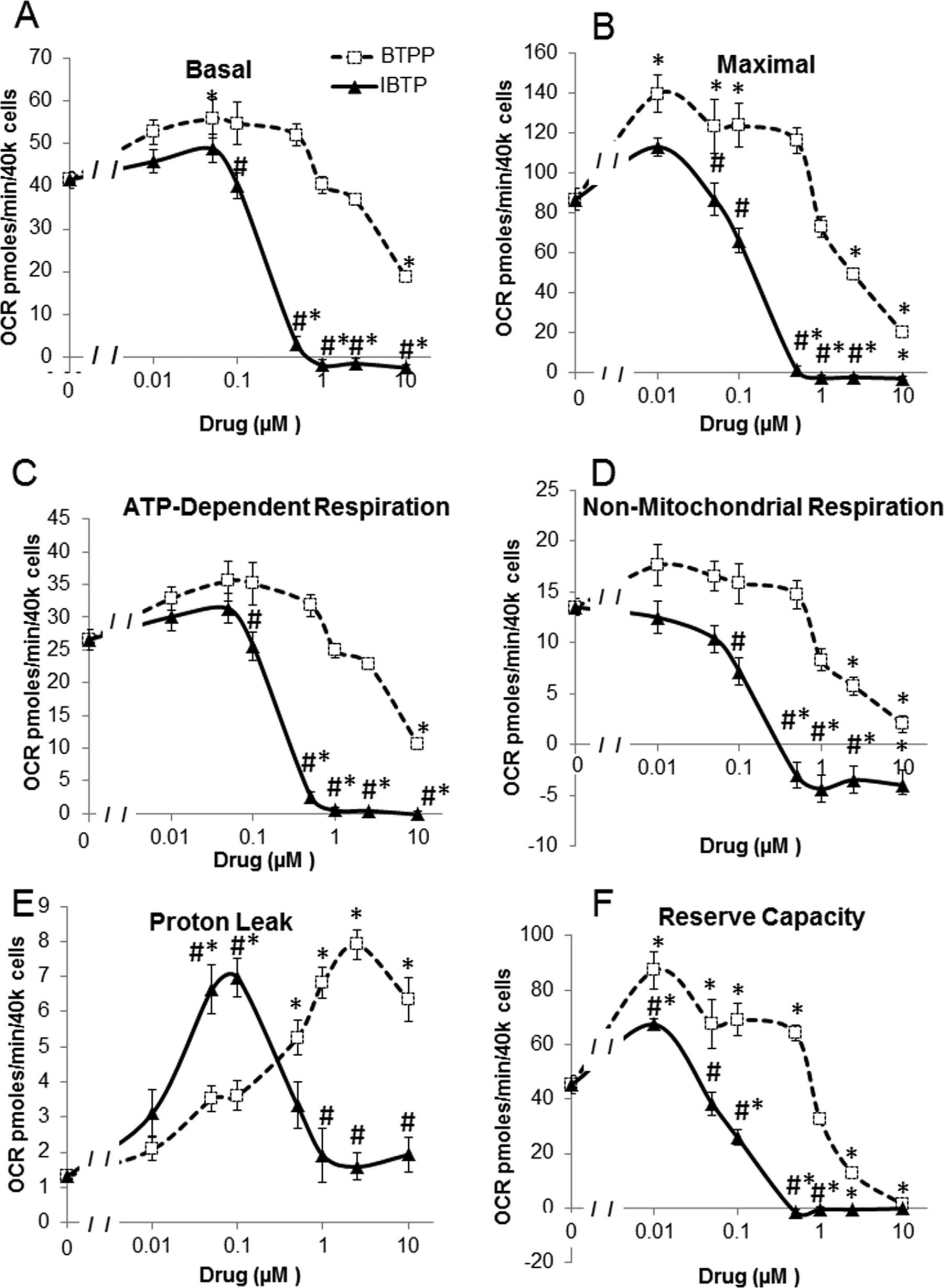
Mitochondrial stress test dose response curves. MCF-10A cells were treated with vehicle (EtOH), IBTP (0.0–10 µM), or BTPP (0.01–10 µM) for 24 h and a mitochondrial stress test was performed as described in the Methods. Panel A: Dose response of IBTP or BTPP on basal respiration. Panel B: Dose response of IBTP or BTPP on maximal respiration. C: Dose response of IBTP or BTPP on ADP-dependent respiration D: Dose response of IBTP or BTPP on non-mitochondrial respiration. E: Dose Response of IBTP or BTPP on proton leak F: Dose response of IBTP or BTPP on reserve capacity. Values are mean ±SEM obtained from 13 to 26 wells in three separate experiments; *P<0.05 compared to vehicle; # *P*<0.05 compared to BTPP.

In order to determine whether IBTP elicited these effects in a non-cancer cell line, we utilized the immortalized epithelial cell line MCF-10A utilizing the same dose range of BTPP and IBTP from above (0.01–10 µM) and the results are shown in Fig. 2. Mitochondrial thiol modification was also found to also decrease basal respiration, maximal respiration, ATP-dependent respiration, non-mitochondrial respiration, proton leak, and reserve capacity in MCF-10A cells at concentrations form 0.1 µM and above compared to vehicle (also indicated as 0 µM) and also exhibited differential effects from BTPP (Fig. 2A–F) Interestingly at lower concentrations IBTP increased proton leak at 0.05 and 0.1 µM concentrations then subsequently dedcreased again. BTPP also exhibited significant decreases in all of the above parameters at the highest concentrations (2.5 and 10 µM) while also exhibiting significant increases in basal respiration, maximal respiration proton leak, and reserve capacity at concentrations 0.01–2.5 µM. These results indicate the ability of IBTP to significantly alter bioenergetics is due to a complex integration between its ability to modify particular protein thiolates and the effects on mitochondrial function of its targeting moiety in both tumorigenic and non-tumorigenic cells.

**Fig. 3:**
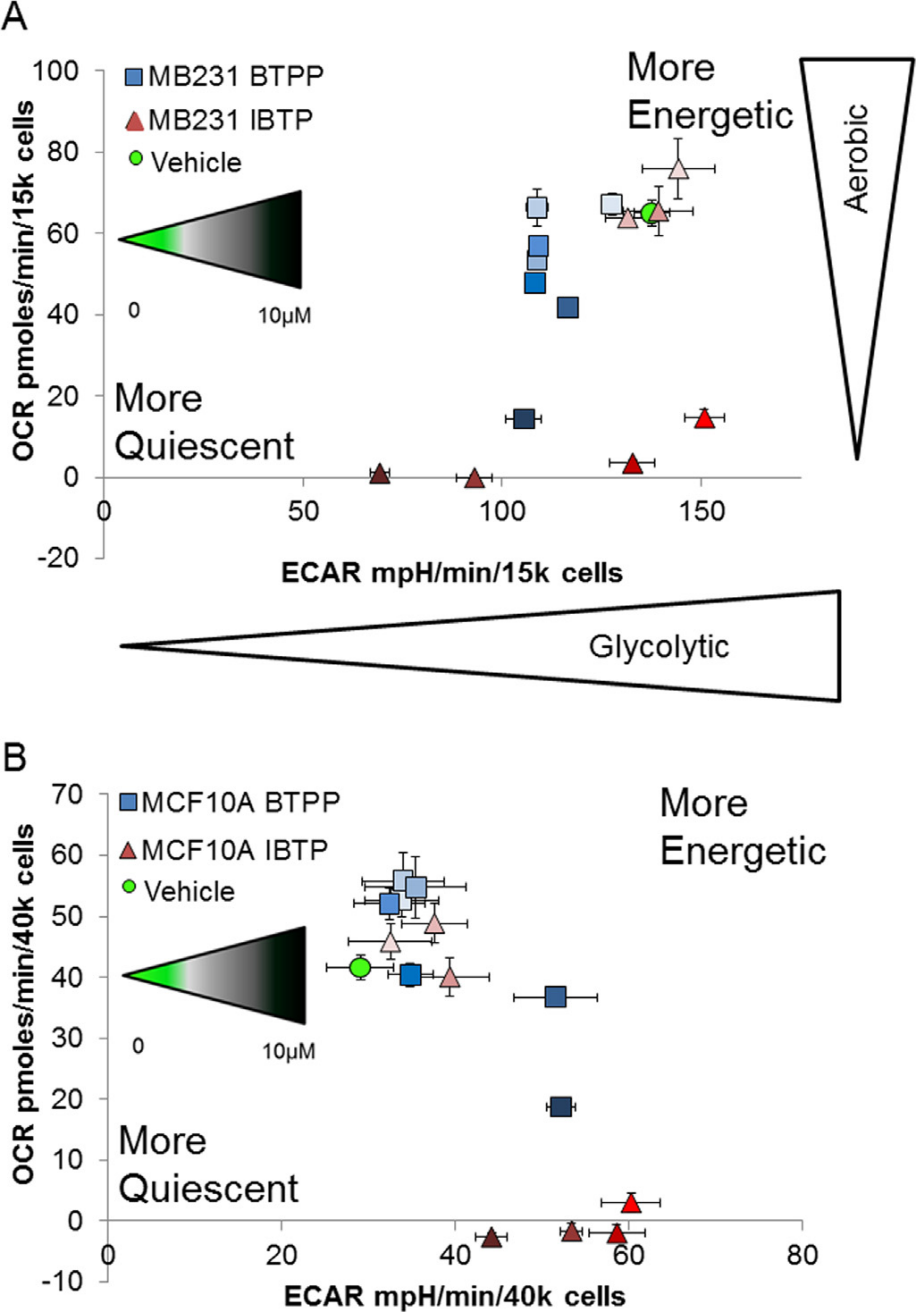
XF Metabolic Phenogram. Panel A: Basal OCR and ECAR rates were plotted in response to a 24h treatment of vehicle (EtOH; ), IBTP (0.01 µM; , 0.05 µM; , 0.1 µM; , 0.5 µM; , 1 µM; , 2.5 µM; , and 10 µM; ) or BTPP (0.01 µM; , 0.05 µM; , 0.1 µM; , 0.5 µM; , 1 µM; , 2.5 µM; , and 10 µM; ) in MB231 cells. Panel B: Basal OCR and ECAR were plotted plotted in response to a 24 h treatment of in MCF-10A cells using the concentrations and values from above. Values are mean±SEM obtained from 11 to 26 wells in 3 separate experiments.

In order to determine how mitochondrial modification by IBTP shifts overall cellular metabolism as concentrations increase, basal OCR which is indicative of OXPHOS was then plotted as a function of basal ECAR (Fig. S1), which is assumed to be representative of glycolytic function to form a bioenergetic phenogram in to determine the overall metabolic phenotype of the cells (Fig. 3). In both cell types, mitochondrial thiol modification did not elicit any effects on bioenergetics at lower concentrations (0–0.1 µM) after modification of IBTP; however as concentrations of IBTP increased (0.5–1 µM) a shift from the energetic quadrant to a more glycolytic state was seen in the MB231 cells indicating a perturbation of oxidative phosphorylation (OXPHOS). At higher concentrations of IBTP (2.5–10 µM) the MB231 cells shifted into the quiescent state indicating perturbations in both OCR and ECAR indicating disruptions in both OXPHOS and glycolytic paradigms respectively (Fig. 3A). BTPP also elicited a shift to a glycolytic state at higher concentrations (1–10 µM) with loss of OCR function, but did not significantly decrease ECAR to the same extent as IBTP (Fig. 3A). In MCF10A cells, a significant decrease in OCR was also seen at concentrations of 0.5–10 µM IBTP, indicating a similar loss of OXPHOS capability in non-tumorigenic cells. However, in contrast to MB231 cells, IBTP significantly increased ECAR at concentrations from 0.5 to 2.5 µM then lowered basal ECAR to baseline at 10 µM, indicating that IBTP effects on ECAR are different between these two cell types. BTPP showed slight inhibitory effects on OCR in both cell lines which became more pronounced at higher concentrations (e.g. 2.5–10 µM); whereas, BTPP caused a compensatory increase in ECAR at these higher concentrations in MCF10A cells only (Fig. 3B). These results indicate that mitochondrial thiol modification alters the metabolic phenotype in both MB231 and MCF10A cells, and the ability of these cells to compensate for the loss of OXPHOS via glycolysis is likely to be dependent on the basal energetic requirements of the cell type. Additionally, higher energetic cells, like MB231 appear to shift these to a more quiescent energetic state after 24 h exposure to IBTP. Overall, these results suggest that IBTP may have differential metabolic effects according to cell type.

### 3.2 Mitochondrial thiol modification affects ATP levels

**Fig. 4:**
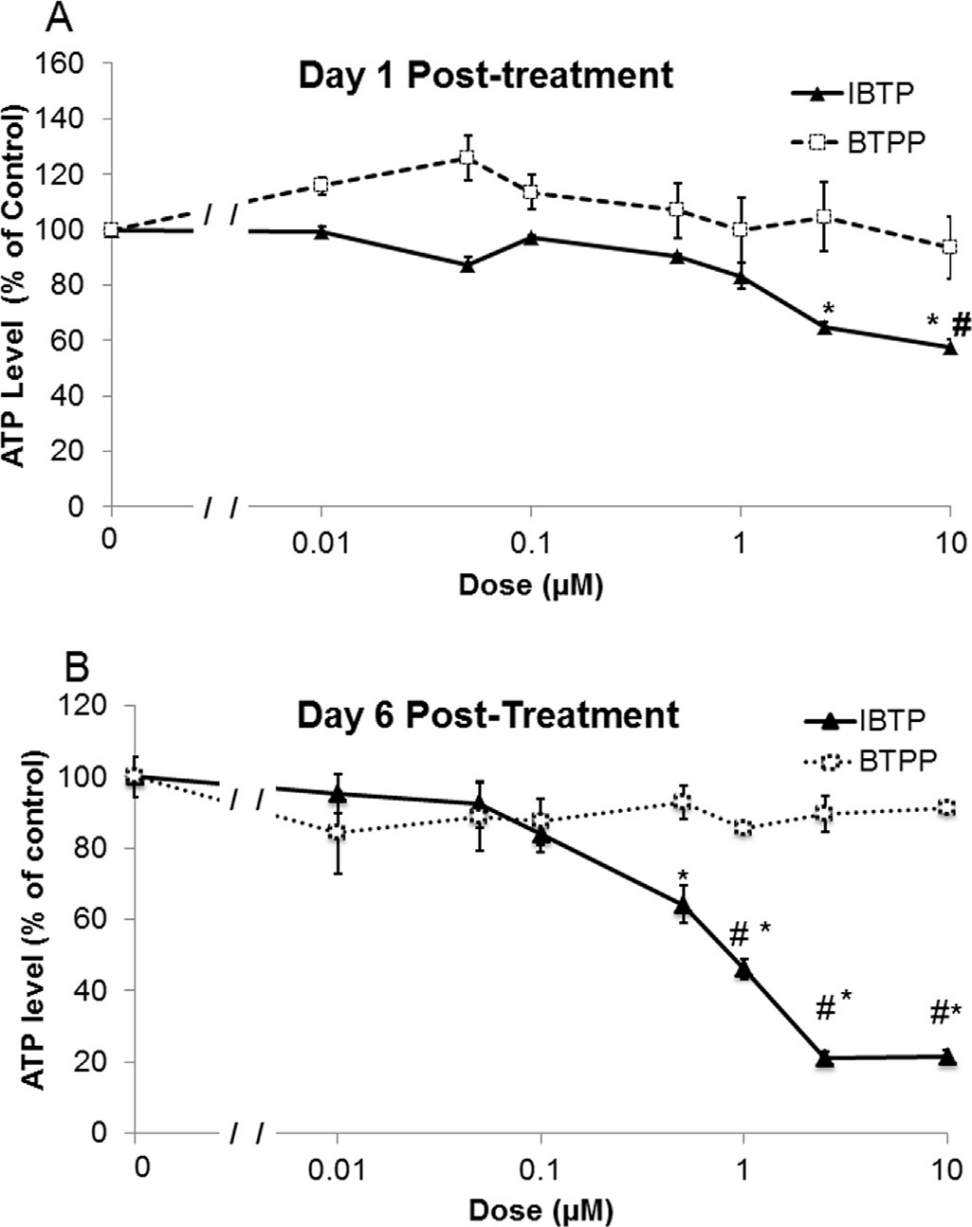
ATP measurements by ATPLite™. Cells were plated on 6-well plates and treated with vehicle (EtOH), IBTP (0.01–10 µM), or BTPP (0.01–10 µM) for 24 h in 0.5% FBS-containing medium. After incubation, medium was changed to 10% FBS-containing medium. Cells were lysed and ATP values were expressed as percent vehicle at each time point Panel A: ATP measurements day 1 post-treatment Panel B: ATP measurements day 6 post-treatment. Values are mean±SEM obtained from 2 separate experiments. *P<0.05 compared to vehicle. #*P*<0.05 compared to BTPP.

Decreased bioenergetic parameters could result in decreased ATP levels; therefore, we measured levels of ATP after mitochondrial thiol modification by IBTP using a luminescence assay. IBTP caused a decrease at higher concentrations (2.5–10 µM) at day 1 post-treatment compared to either vehicle or BTPP. At day 6 post-treatment, ATP levels remained decreased when compared to vehicle and BTPP (0.5–10 µM). BTPP exhibited no changes to ATP at any concentration at either day 1 post-treatment or day 6 post-treatment (Fig. 4A–B).

**Fig. 5:**
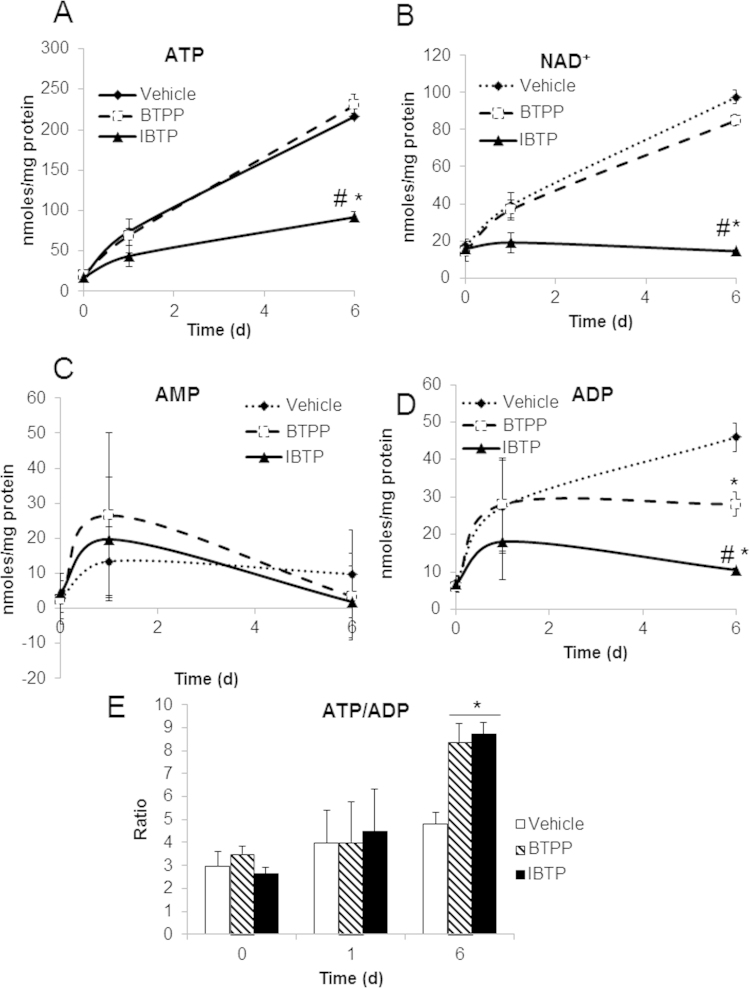
Cellular adenine nucleotide levels. Cells were plated on 6-well plates and treated with vehicle (EtOH), IBTP (10 µM), or BTPP (10 µM) for 24 h in 0.5% FBS-containing medium. After incubation, medium was changed to 10% FBS-containing medium. At days 0, 1, and 6, cells from one 6-well plate were lysed and adenine nucleotides were measured by HPLC analysis Panel A: ATP levels were expressed as nmol/mg protein. Panel B: NAD^+^ levels were expressed as nmol/mg protein Panel C: AMP levels were expressed as nmol/mg protein. Panel D: ADP levels were expressed as nmol/mg protein. Panel E: Ratio of ATP to ADP at each time point. Values are mean±SEM obtained from 3 separate experiments. *P<0.05 compared to vehicle. #*P*<0.05 compared to BTPP.

**Fig. 6:**
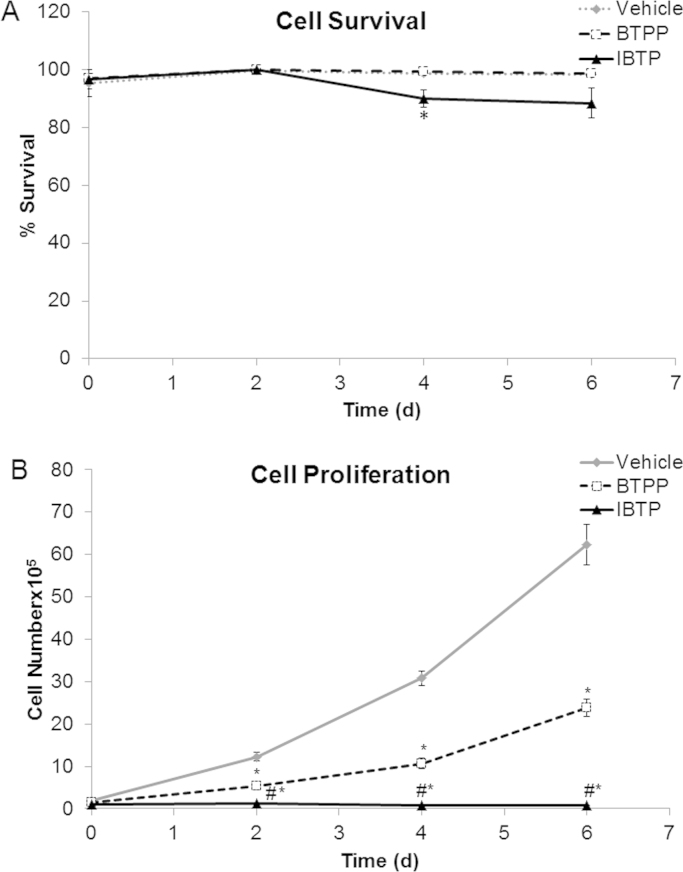
Cell survival and proliferation after mitochondrial thiol modification. Panel A: MB231 cells were treated with vehicle (EtOH), IBTP (10 µM), or BTPP (10 µM) for 24h and cell survival were measured by trypan blue exclusion as described in the methods. Panel B: Cells were treated as above and overall cellular proliferation was measured by counting cells using an automated hemocytometer. The values are mean±SEM, n=3 samples obtained from 1 independent experiment. *P<0.05 vs vehicle, #*P*<0.05 vs BTPP.

Because decreased ATP levels could indicate either altered energetic status or cell death, changes to other key adenonucleotides in response to mitochondrial thiol modification were also analyzed using an HPLC-based technique. Adenonucleotide levels were monitored at days 0, 1 and 6 post-treatment, and IBTP decreased overall ATP to approximately 40% of vehicle control at day 6 post-treatment (Fig. 5A). NAD^+^ levels were also decreased by IBTP compared to vehicle or BTPP **(**Fig. 5B). AMP exhibited no changes at any time points, whereas ADP levels were decreased by BTPP and IBTP at day 6 post-treatment **(**Fig. 5C and D) though the effect of IBTP was more pronounced. The ATP: ADP ratio indicates cellular energy supply and demand, and these values were higher with both BTPP and IBTP at day 6 post-treatment (Fig. 5E). When these data were replotted as % of vehicle control, the relative decrease in adenonucleotides over time is more apparent (Fig. S2), demonstrating that although absolute amounts of adenonucleotides increase over time, the relative levels compared with vehicle at each time point are actually decreased. These results indicate that mitochondrial thiol modification by IBTP in cancer cells causes a sustained reduction in both energetic supply and demand in treated cells. The targeting moiety BTPP also caused a slight decrease in ADP and an increase in the ATP: ADP ratio indicating that the targeting moiety may play a role in decreasing energetic demand. Despite the remarkable decreases in adenonucleotides, cell survival was only slightly decreased at day 4, while no changes to cell survival were observed at day 6 (Fig. 6A). However, overall cell numbers were significantly decreased compared to either vehicle or BTPP **(**Fig. 6B) indicating that cellular proliferation was attenuated by mitochondrial thiol modification. However, significant cell proliferation was observed with BTPP at day 2 and beyond, in contrast to IBTP. These results indicate that the decrease in energetic demand caused by the mitochondrial thiol modification of IBTP does not induce apoptosis but rather switches cells into a more quiescent energetic state and inhibits cellular proliferation.

### 3.3 Mitochondrial thiol modification effects on metabolite levels

**Fig. 7:**
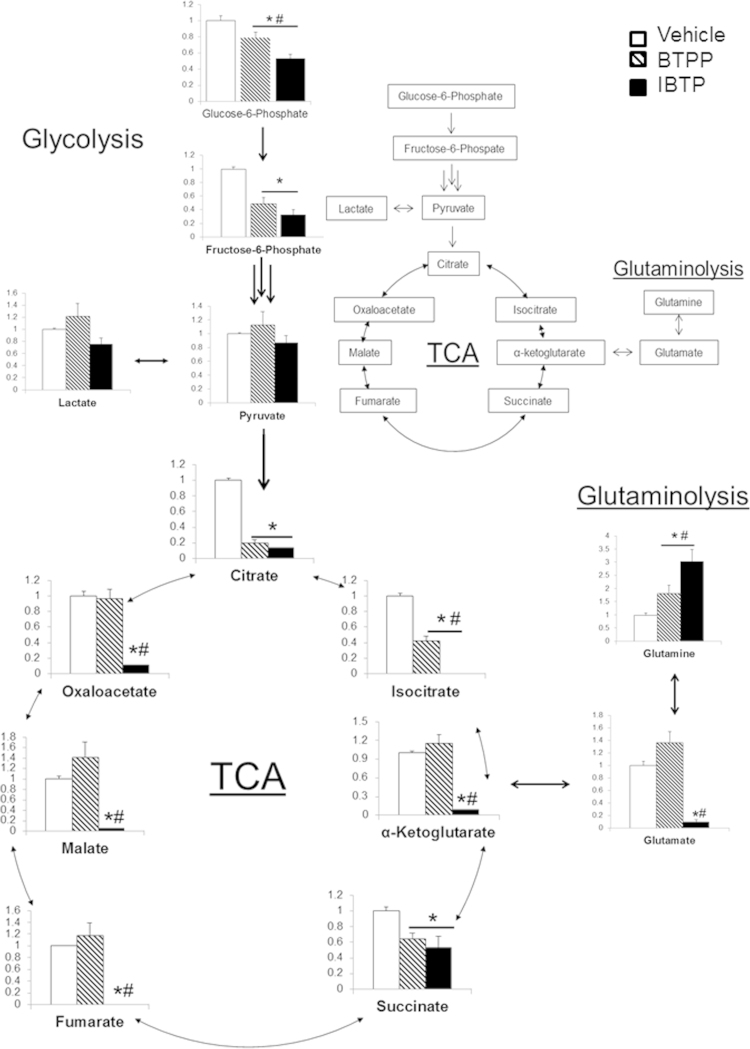
Targeted metabolomic analysis. MB231 cells were treated with vehicle (EtOH), IBTP (10 µM), or BTPP (10 µM) for 24 h and Krebs cycle and related metabolite levels were measured. The values are mean ±SEM, *n*=5 samples *P<0.05 compared to vehicle. #*P*<0.05 compared to BTPP.

In order to determine the effects of mitochondrial thiol modification on glycolysis and the Krebs cycle in MB231 cells, key metabolites were measured in response to IBTP (10 µM) after a 24 h exposure. Mitochondrial thiol modification decreased the glycolytic intermediates glucose-6-phosphate and fructose-6-phosphate when compared to vehicle and BTPP, while both pyruvate and lactate were unchanged. IBTP also decreased Krebs cycle metabolites citrate, α-ketoglutarate, succinate, malate and oxaloacetate (Fig. 7). Isocitrate and fumarate were below the limit of detection in cells treated with IBTP. Additionally, IBTP increased the amino acid glutamine and decreased glutamate, indicating inhibition of the glutaminolysis pathway. BTPP also decreased citrate, isocitrate, succinate, and glutamine, indicating that the targeting moiety has effects on Krebs metabolites, albeit to a lesser extent than IBTP. These results indicate that mitochondrial thiol modification decreases metabolites within the Krebs cycle and anaplerotic pathways feeding into the Krebs cycle, including glutaminolysis and glycolysis.

### 3.4 Mitochondrial thiol modification effects on aconitase

**Fig. 8:**
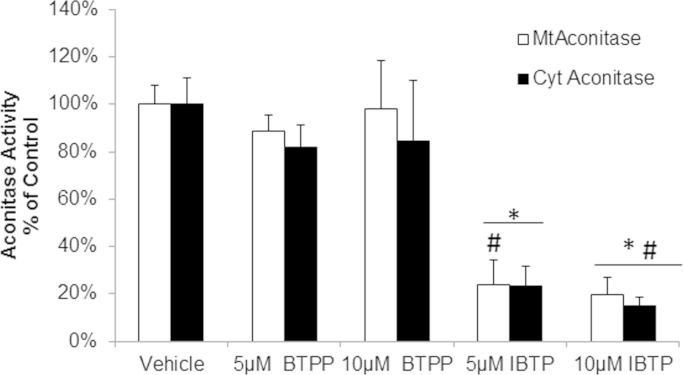
Thiol Modification of Aconitase Activity. MB231 cells were treated with IBTP (5–10 µM) or BTPP (5–10 µM) for 24 h. Cells lysates were prepared and aconitase activity was measured via in-gel activity assay. Values represent mean±SEM, *n*=4 obtained from 2 independent experiments; *P<0.05 compared to vehicle. #*P*<0.05 compared to BTPP.

The fact that Krebs cycle metabolites were decreased with mitochondrial thiol modification suggests inhibition of substrate entry at one or more key regulatory enzymes. Aconitase (ACO) was previously identified as an IBTP target protein; therefore, its activity was determined in response to IBTP [25], [28]. An in-gel activity assay was used to monitor both cytosolic and mitochondrial aconitase activities in response to EtOH vehicle, BTPP, and IBTP (5 or 10 µM) for 24 h. Mitochondrial aconitase activity was decreased after treatment with IBTP at both concentrations when compared to vehicle and BTPP; however cytosolic aconitase was decreased only at the highest concentration (10 µM) of IBTP (Fig. 8). BTPP exhibited no effects on either isoform of aconitase at any of the concentrations used. These results indicate that mitochondrial thiol modification decreases activity of aconitase and that one of the first committed steps in the Krebs cycle is regulated by thiol status. Abundance of cytosolic aconitase protein was measured by western blot and no changes in protein level were observed in response to either IBTP or BTPP; while mitochondrial aconitase was below the limit of detection (data not shown).

### 3.5 Mitochondrial thiol modification effects on GAC

**Fig. 9:**
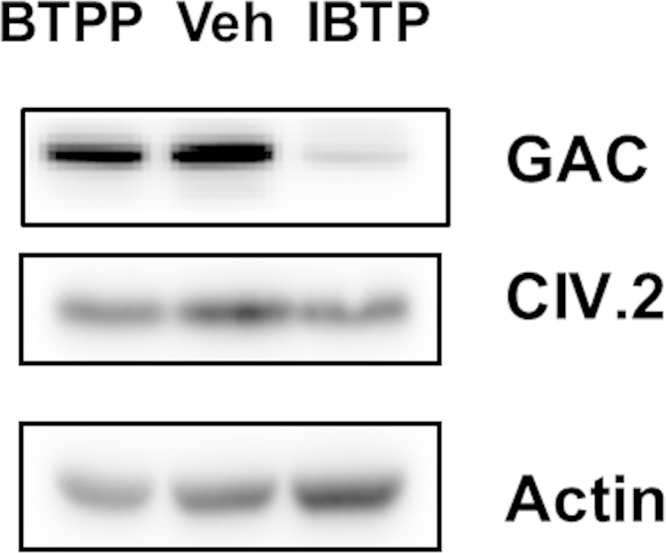
Glutaminase and other protein levels. MB231 cells were treated with vehicle (EtOH), IBTP (10 µM), or BTPP (10 µM) for 24 h. Cell lysates were prepared and proteins were visualized by Western blot analysis using a polyclonal anti-GAC/KGA antibody, complex IV subunit 2 (CIV.2), or β-actin. The images within each protein were taken from the same blot image and identically contrasted for representation. Images are representative from two independent experiments.

Glutaminolysis in many cancer cells is mediated by glutaminase C (GAC), a splice variant of kidney-type glutaminase (KGA) which is thought to possess a reactive thiol group [17]. As previously shown in Fig. 6, mitochondrial thiol modification increased glutamine levels and decreased glutamate levels indicating inhibition of the glutaminolysis pathway. Therefore, the effects of the IBTP on GAC protein levels were monitored. MB231 cells were treated for 24 h with EtOH vehicle, IBTP or BTPP (10 µM), and abundance of GAC was determined by western blot (Fig. 9). Mitochondrial thiol modification by IBTP decreased GAC protein levels, compared to vehicle and BTPP, which was found to have had no effect on GAC levels. Additionally, IBTP had no effect on levels of other mitochondrial and cytosolic proteins including complex IV subunit 2, and β-actin indicating that general protein degradation or autophagy are not causing the decrease in GAC and that the decrease in GAC protein levels is modulated by thiol modification.

**Fig. 10:**
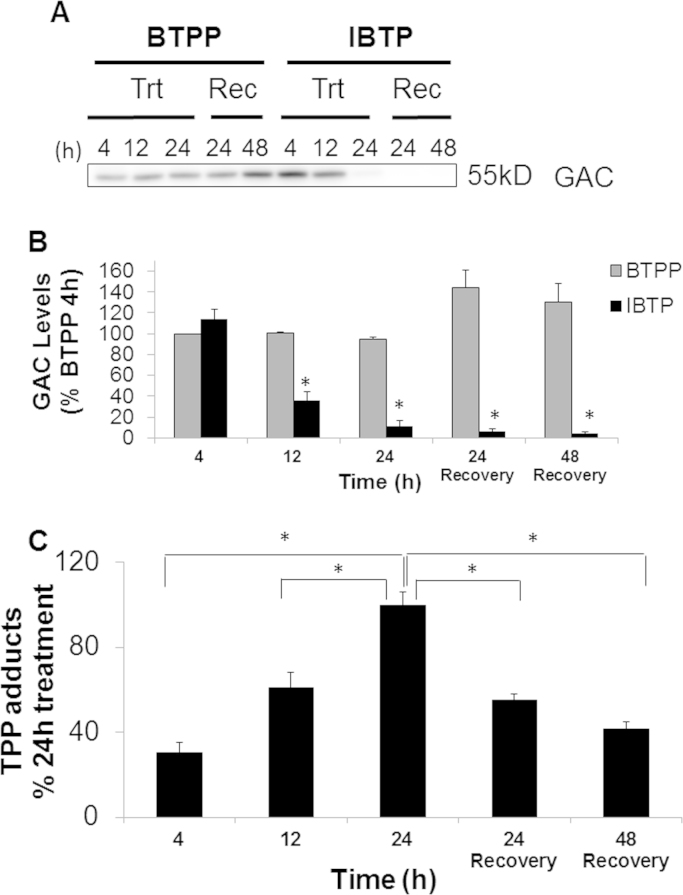
Time course of glutaminase protein levels. Panel A: MB231 cells were treated with IBTP (10 µM) or BTPP (10 µM) for the indicated times (4–24 h), or were treated for 24 h followed by removal of the treatment and recovery for 24 or 48 h. Representative image of western blots for glutaminase (GAC and KGA) obtained using a polyclonal anti-GAC/KGA antibody. Cytosolic aconitase (ACO1) was used as a loading control**.** Panel B: Quantification of GAC from Panel A. Values represent mean±SEM, *n*=9 obtained from three independent experiments; *P<0.05 compared to 4hr BTPP treatment. Panel C: TPP adducts in IBTP-treated cell lysates were visualized by Western blot analysis and quantified. Values represent percent of adducts observed at 24 h. Values represent mean ±SEM of 9 samples obtained from three independent experiments; *P<0.05 compared to 24 h IBTP treatment.

The effects of IBTP on GAC protein levels over time were then studied. Cells were treated with either BTPP or IBTP (10 µM) for various time points and GAC protein levels were monitored. BTPP had no effect on GAC as seen in Fig. 8 while IBTP treatment decreased GAC protein levels to 36% of BTPP levels after 12h and to 12% of BTPP levels after 24h (Fig. 10A and B). When cells were treated for 24 h and allowed to recover up to 48 h in drug free media, GAC protein levels did not recover, but remained near 5% of BTPP levels (Figs. 10A and B). To ensure that the decrease in GAC protein abundance was not due to disruption of an epitope on GAC after TPP adduct formation, the decrease in GAC was confirmed using a different antibody (Fig. S3). Overall, these results indicate that GAC protein levels are decreased in response to mitochondrial thiol modification, and that this effect persists after removal of IBTP.

We then sought to determine whether the decrease in GAC protein corresponds temporally to the accumulation of TPP adducts. Cell lysates were probed for TPP-protein adducts by Western blot analysis, and were significantly increased over the time-frame corresponding to the GAC protein decrease (Fig. 10C). However, when cells were allowed to recover in drug-free medium, TPP adducts decreased by nearly half, but remained as high as 42% of maximal up to 48 h after removal of IBTP. Interestingly, some individual TPP-protein adduct bands were profoundly decreased, while others appeared stable (Fig. S4), open arrowheads vs. closed arrowheads). As expected, protein adducts were not observed in cells treated with the non-electrophilic analog BTPP (Fig. S4). These results demonstrate that TPP adducts are formed over a similar time frame as GAC protein depletion, and suggest that the fates of TPP-adducts are protein-dependent, resulting in TPP adduct clearance, changes in protein level, or persistence of adducts on specific proteins.

## 4 Discussion

In this study, we examined the functional effects of mitochondrial thiol modification through the use of a mitochondria-targeted electrophile, IBTP, metabolism, and enzyme activity and protein levels using both a breast cancer cell line (MB231) and an immortalized nontumorigenic epithelial cell line (MCF-10A). Other TPP-linked compounds including mitochondria-targeted ubiquinol (MitoQ), and mitochondria-targeted α-tocopherol (MitoE) have previously been shown to inhibit mitochondrial respiration, and other studies including our own have indicated that TPP^+^ itself can elicit effects on mitochondrial bioenergetics [27], [34]. Length of the TPP^+^ moiety has also been shown to a factor in the inhibition of mitochondria metabolism. In fact, increasing the linker portion of the targeting moiety to 8 carbons or higher was shown to suppress mitochondrial bioenergetics and dysregulate mitochondrial calcium channel function. The effects of longer chain linker regions could lead to a more pro-cytotoxic environment when compared to shorter chain TPP^+^-linked compounds such as IBTP, which has a 4 carbon linker. The role of IBTP and other shorter chain TPP^+^ compounds in calcium dysregulation have not been elucidated to date [35], [36], [37], We showed that both BTPP and IBTP elicited effects on bioenergetic parameters in both cell lines (Fig. 1, Fig. 2), but that IBTP, which is capable of thiol modification, was more potent than BTPP, which contains only the targeting moiety and a non-reactive alkyl group, suggesting that thiol modification contributes to inhibition of mitochondrial respiration. We determined that the decrease in bioenergetics was not due to a decrease in overall mitochondrial content, since levels of citrate synthase protein were not changed after exposure to the highest concentration of BTPP or IBTP (10 µM; data not shown). The fact that non-mitochondrial respiration was significantly decreased by IBTP is also of interest. Non-mitochondrial respiration has a number of potential sources of oxygen consumption including xanthine oxidase, cytochrome P450 and other forms of oxidases [38], [39]. Interestingly, xanthine synthesis requires adenonucleotides, which we have shown to be decreased after IBTP treatment, in order to generate the precursor inosine. Decreased inosine in turn would lead to decreased hypoxanthine, which is a substrate of xanthine oxidase. While other oxygen consuming cytosolic pathways could be involved, this is an example of one potential pathway which could explain the effect of mitochondrial thiol modification on non-mitochondrial respiration.

Interestingly, the metabolic phenograms of both cell lines show that, at baseline, the MB231 cells are in a higher energetic state than the non-tumorigenic MCF10A cells (Fig. 3A and B), and by treating with increasing concentrations of IBTP a significant decrease in both OCR and eventually ECAR leading to a more quiescent state in MB231 cells is seen. However, in the MCF10A cells, a significant compensatory increase in ECAR occurs simultaneously with the decrease in OCR at all but the highest concentration which appears to exceed the cells compensatory mechanisms. The increase in ECAR could be a result of increased glycolysis, and could represent the cell's attempt to maintain homeostasis and adequate ATP levels. Due to the differential metabolic effects of IBTP on tumorigenic and non-tumorigenic cells, mitochondria-targeted thiol modification could have significant therapeutic potential; however, additional studies are warranted to investigate this possibility.

Adenonucleotide amounts increased over time, even after normalization to protein amounts (Fig. 5A–D). Dolfi et al. showed ATP levels per cell are not constant, but are proportional to cell size [40]. Thus, we speculate that ATP levels increase due to changes in cell size or volume over 6 days. Another key difference observed in IBTP-treated cells which was not recapitulated in BTPP-treated cells, was the induction of a more quiescent energetic state. This was supported by a decrease in the overall ATP: ADP ratio, indicating a lower steady-state for cellular energy supply and demand (Fig. 5E and S2) [41], [42], [43]. This increase in ATP: ADP could also indicate a shift to a higher glycolytic state, a phenomenon which has been previously reported [44] and is supported by our ECAR data (Fig. S1). Perhaps one of the most surprising results in our study was that no appreciable cell death was observed in IBTP-treated cells at the highest concentration of compound tested (10 µM), despite the profound alterations in bioenergetics and adenonucleotides (Fig. 6A). However, the decrease in bioenergetics and adenonucleotides may have contributed to the decrease in cellular proliferation in MB231 cells, since they exhibited a more quiescent energy state in response to IBTP adduction in the metabolic phenograms (Fig. 6B). It is not clear whether this shift toward a quiescent energy state is equivalent to true cellular quiescence, or whether the cells are slowly undergoing cell death. Although some cancer cells can remain quiescent for long periods of time, further studies are necessary to determine the nature of IBTP-induced quiescence and how long it can persist *in vivo*.

The decreased energy status of IBTP-treated cells was accompanied by decreased glycolytic and Krebs cycle metabolites, suggesting an upstream role for mitochondrial protein thiol modification in metabolite flux leading to energy production (Fig. 7). Interestingly, the fact that IBTP decreases glycolytic metabolites was unexpected. While we cannot rule out the possibility that IBTP forms a small of number of cytosolic adducts that could inhibit glycolysis, the rate of adduct formation of iodo-compounds with thiols is relatively slow compared with the rate of TPP^+^-accumulation within the mitochondria [45], which would not support the concept of cytosolic protein adducts being a major factor. In addition, glucose-6-phosphate and fructose-6-phosphate levels were decreased by both BTPP and IBTP, suggesting that the effects on glycolysis are not due to thiol modification, but are more likely due to the TPP^+^ moiety. Nevertheless, the effects of IBTP and BTPP on glycolysis could be due to either direct interaction of the compounds with cytosolic components, or indirect action of these compounds on mitochondrial calcium channels or other processes. The fact that ADP levels are decreased by both IBTP and BTPP suggests that allosteric activation of glycolysis may be suppressed by these compounds. Other possibilities include increased flux through the pentose-phosphate pathway which could shunt glucose-6-phosphate from glycolysis in order to generate NADPH.

Changes to the Krebs cycle metabolites α-ketoglutarate, fumarate, malate, and oxaloacetate were not observed with BTPP treatment, indicating that the anti-anaplerotic effects are dependent on mitochondrial thiol modification. Previous studies by our lab and others using isolated mitochondria in other cell types have shown that IBTP forms adducts with several proteins involved in the Krebs cycle [24], [25], [28], [29]. In this study, we showed that aconitase enzymatic activity and GAC protein levels were decreased by IBTP (Fig. 8, Fig. 9, Fig. 10). These enzymes represent two important entry points for metabolic intermediates into the Krebs cycle, and metabolites downstream of these enzymes were decreased in cells exposed to IBTP. In addition, other metabolites were also decreased, presumably because intermediates were not available for conversion, and/or because other Krebs cycle enzymes are inhibited by IBTP which have not previously been reported. Aconitase has previously been shown to be a target of IBTP adduction, but it is not yet known whether GAC is also adducted. In a study by Campos et al., N-ethylmaleimide (NEM), a thiol-reactive reagent, inhibited glutaminase activity. Further studies will be necessary to determine whether there is a reactive cysteine near or within the binding site of GAC which could be modified by IBTP [17]. The fact that IBTP decreased GAC protein, without affecting the levels of other proteins such as complex IV subunit 2, or β-actin, suggest that GAC protein is selectively decreased by mitochondrial thiol modification (Fig. 9, Fig. 10). It is important to note that GAC's role in glutaminolysis is to convert glutamine to glutamate which can then be converted to either α-ketoglutatate, or other amino acids such as alanine, aspartate, or arginine which are required for protein synthesis. With the loss of GAC, we would expect that the amino acid substrates required protein would become limited and this could potentially result in premature termination of protein synthesis. The decrease in GAC protein is time-dependent and unrecoverable up to 48 h after IBTP was removed (Figs. 10A and B; S3). However, the exact mechanism by which mitochondrial thiol modification decreases GAC protein levels remains to be elucidated.

In conclusion, we have demonstrated that mitochondrial thiol modification by IBTP induces a type of metabolic reprogramming resulting in decreased mitochondrial bioenergetics, Krebs cycle metabolites and adenine nucleotides in MB231 breast cancer cells. The data presented in this study suggest mitochondrial thiol modification regulates metabolism and inhibits anaplerosis by decreasing aconitase enzyme activity and GAC protein levels. These effects on metabolism by mitochondrial thiol modification have important implications for the basic understanding of cancer cell metabolism and the development of thiol specific cancer therapeutics.

## Author contributions

M. Ryan Smith, Praveen K. Vayalil, Fen Zhou, Gloria A. Benavides, and Aimee Landar conceived and designed the experiments. M. Ryan Smith, Fen Zhou, Praveen K. Vayalil Gloria A. Benavides, Reena Beggs, Hafez Golzarian, Bhavitavya Nijampatnam, Patsy G. Oliver performed the experiments. M. Ryan Smith, Gloria A. Benavides, Patsy G. Oliver, Sadanandan E. Velu, and Aimee Landar analyzed the data. M. Ryan Smith and Aimee Landar wrote the manuscript. All authors participated in the preparation and editing of the manuscript.

## Declarations of interest

A.L., S.E.V., M.P.M., and R.A.J.S. have a pending patent application (serial number PCT/US2014/051372) for the mitochondria-targeted electrophilic compounds in this study and related mitochondria-targeted electrophilic compounds, and their use in cancer. This did not influence the results or interpretations of this study in any way.

## Appendix A Supplementary material

Supplementary material **Figure S1: Basal ECAR of MB231 and MCF10A cells in response to BTPP or IBTP.** MB231 cells were treated with vehicle (EtOH), IBTP (0.01-10 µM), or BTPP (0.01-10 µM) for 24 h and basal ECAR was measured in parallel with the mitochondrial stress tests described in Fig. 1, Fig. 2. **Panel A:** Dose response of IBTP or BTPP on basal ECAR in MB231 cells. **Panel B:** Dose response of IBTP or BTPP on basal ECAR in MCF10A cells. Values are mean ±SEM obtained from 11-26 wells in three separate experiments; *P <0.05 compared to vehicle; # P <0.05 compared to BTPP.

Supplementary material **Figure S2: Adenine nucleotide levels replotted as % of control.** Adenine nucleotide levels measured by HPLC analysis from Fig. 5 are replotted, and values are expressed as % of vehicle control at each time point ±SEM obtained from 3 separate experiments. **P*<0.05 compared to vehicle and #*P*<0.05 IBTP compared to BTPP.

Supplementary material **Figure S3: Validation of IBTP effects on GAC protein levels.** MB231 cells were treated with IBTP (10 µM) or BTPP (10 µM) for the indicated times (4-24h), or were treated for 24 h followed by removal of the treatment and recovery for 24 or 48 h. **Panel A:** Image from Western blot analysis using a monoclonal anti-GAC/KGA/GAM antibody**. Panel B:** Quantification of GAC from Panel A. Values represent mean ±SD, n=2 from one independent experiment.

Supplementary material **Figure S4: Time course of TPP adduct formation.** MB231 cells were treated with either IBTP (10 µM) or BTPP (10 µM) for the indicated times. Cell lysates were prepared and protein adducts were visualized by Western blot analysis using an antibody directed against the TPP moiety (1:3000). Open arrows indicated by the ˃ symbol show an increase in TPP adducted proteins and a subsequent decrease of that protein over time. Closed arrows, indicated by the ► symbol, indicate protein that are stable and do not appear to change over time.
