# Supplementary figures and images for: Mitochondrial thiol modification by a targeted electrophile inhibits metabolism in breast adenocarcinoma cells by inhibiting enzyme activity and protein levels
Source: Redox Biol. 2016 Jan 8;8:136–48. doi: 10.1016/j.redox.2016.01.002 (PMC4732023; doi:10.1016/j.redox.2016.01.002)

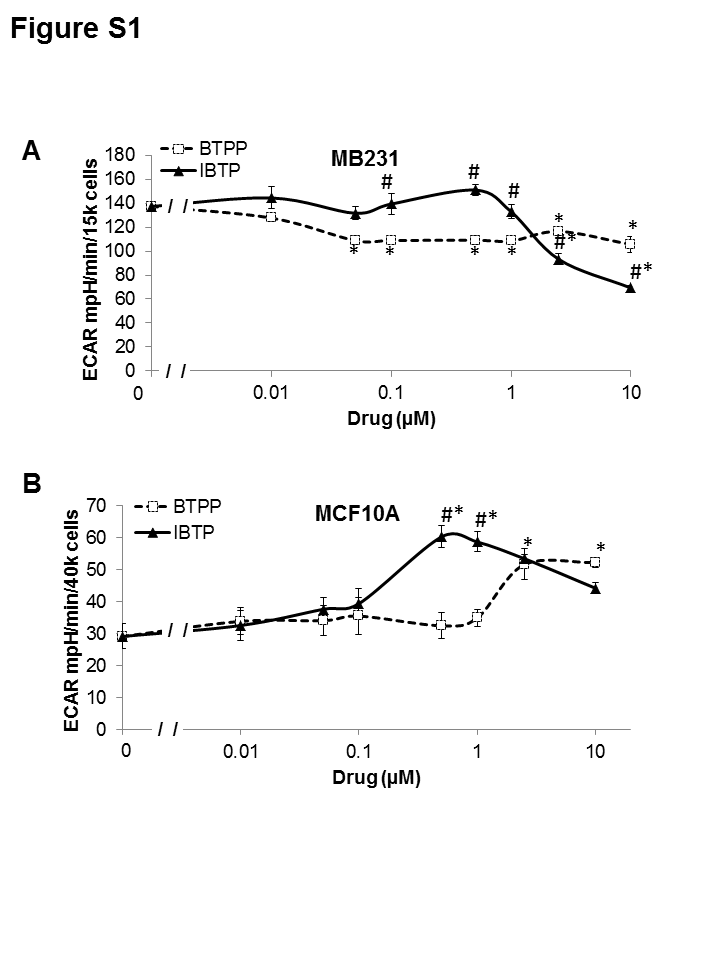

Supplement: Supplementary file 1 — Supplementary material Figure S1: Basal ECAR of MB231 and MCF10A cells in response to BTPP or IBTP. MB231 cells were treated with vehicle (EtOH), IBTP (0.01-10 µM), or BTPP (0.01-10 µM) for 24 h and basal ECAR was measured in parallel with the mitochondrial stress tests described in Fig. 1, Fig. 2. Panel A: Dose response of IBTP or BTPP on basal ECAR in MB231 cells. Panel B: Dose response of IBTP or BTPP on basal ECAR in MCF10A cells. Values are mean ±SEM obtained from 11-26 wells in three separate experiments; *P <0.05 compared to vehicle; # P <0.05 compared to BTPP. [file mmc1.zip › SlideS1.TIF]

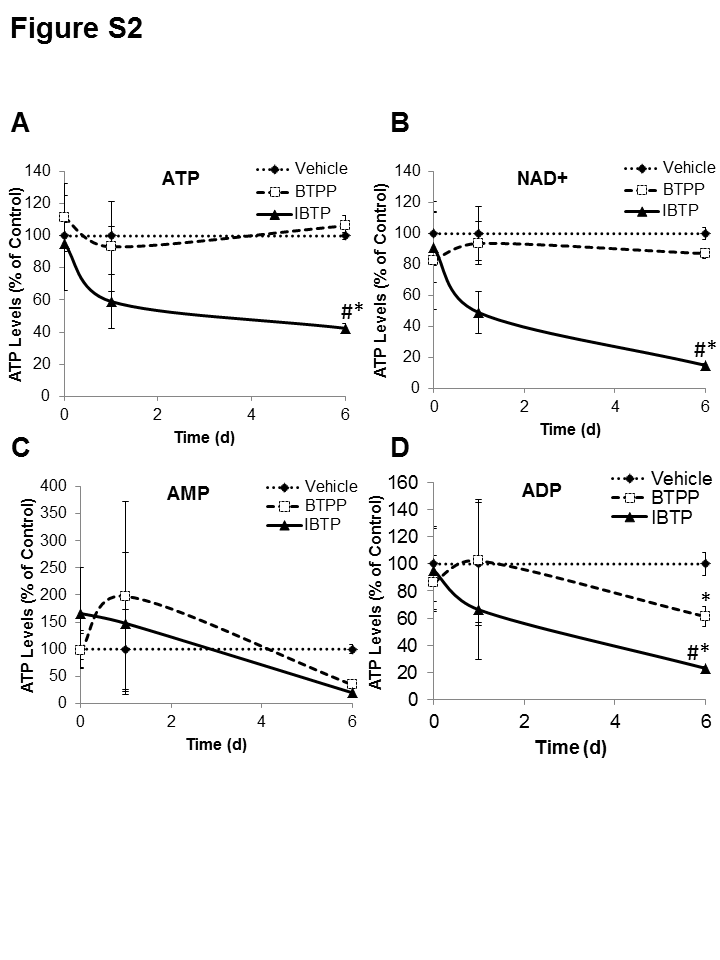

Supplement: Supplementary file 2 — Supplementary material Figure S2: Adenine nucleotide levels replotted as % of control. Adenine nucleotide levels measured by HPLC analysis from Fig. 5 are replotted, and values are expressed as % of vehicle control at each time point ±SEM obtained from 3 separate experiments. *P<0.05 compared to vehicle and #P<0.05 IBTP compared to BTPP. [file mmc2.zip › SlideS2.TIF]

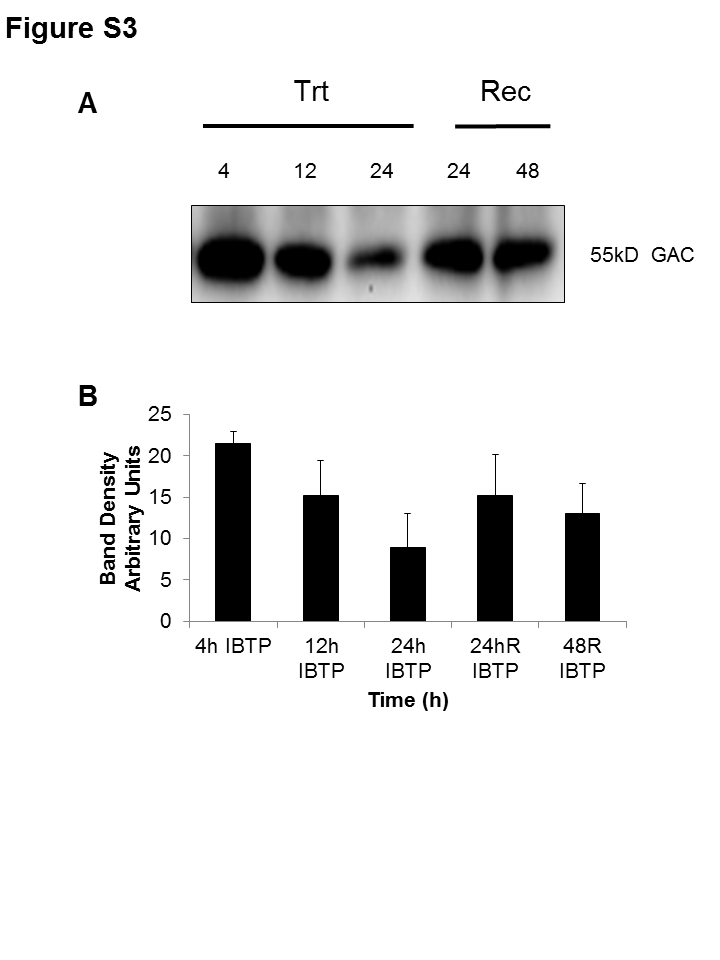

Supplement: Supplementary file 3 — Supplementary material Figure S3: Validation of IBTP effects on GAC protein levels. MB231 cells were treated with IBTP (10 µM) or BTPP (10 µM) for the indicated times (4-24h), or were treated for 24 h followed by removal of the treatment and recovery for 24 or 48 h. Panel A: Image from Western blot analysis using a monoclonal anti-GAC/KGA/GAM antibody. Panel B: Quantification of GAC from Panel A. Values represent mean ±SD, n=2 from one independent experiment. [file mmc3.zip › SlideS3.TIF]

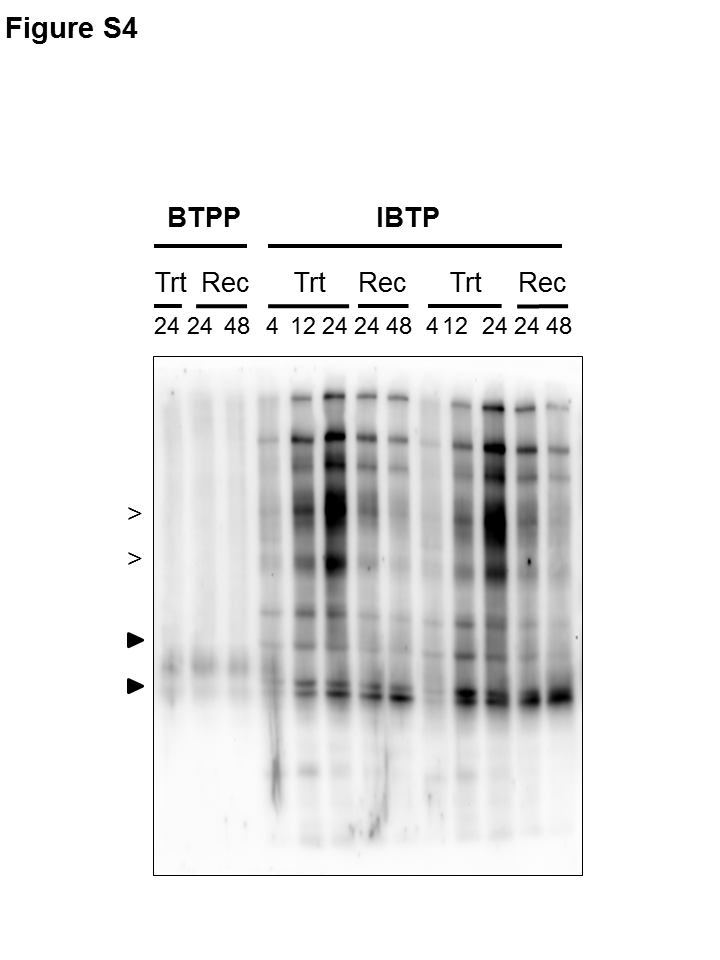

Supplement: Supplementary file 4 — Supplementary material Figure S4: Time course of TPP adduct formation. MB231 cells were treated with either IBTP (10 µM) or BTPP (10 µM) for the indicated times. Cell lysates were prepared and protein adducts were visualized by Western blot analysis using an antibody directed against the TPP moiety (1:3000). Open arrows indicated by the ˃ symbol show an increase in TPP adducted proteins and a subsequent decrease of that protein over time. Closed arrows, indicated by the ► symbol, indicate protein that are stable and do not appear to change over time. [file mmc4.zip › SlideS4.TIF]
